# Review on mushroom mycelium-based products and their production process: from upstream to downstream

**DOI:** 10.1186/s40643-024-00836-7

**Published:** 2025-01-10

**Authors:** Hyun-Jae Shin, Hyeon-Su Ro, Moriyuki Kawauchi, Yoichi Honda

**Affiliations:** 1 Department of Biochemical Engineering, Chosun University, Gwangju, Republic of Korea; 2 Department of Bio and Medical Big Data (BK4 Program) and Research Institute of Life Sciences, Gyeongsang National University, Jinju, Republic of Korea; 3 Laboratory of Environmental Interface Technology of Filamentous Fungi, Graduate School of Agriculture, Kyoto University, Kyoto, Japan; 4 Laboratory of Forest Biochemistry, Graduate School of Agriculture, Kyoto University, Kyoto, Japan

**Keywords:** Mushroom, Mycelium, Biomaterials, Biocomposite, Mycofabrication, Upstream, Downstream, Molecular breeding, Valorization, Bioproducts

## Abstract

**Abstract:**

The global trend toward carbon neutrality and sustainability calls for collaborative efforts in both the basic and applied research sectors to utilize mushroom mycelia as environmentally friendly and sustainable materials. Fungi, along with animals and plants, are one of the major eukaryotic life forms. They have long been utilized in traditional biotechnology sectors, such as food fermentation, antibiotic production, and industrial enzyme production. Some fungi have also been consumed as major food crops, such as the fruiting bodies of various mushrooms. Recently, new trends have emerged, shifting from traditional applications towards the innovative use of mushroom mycelium as eco-friendly bioresources. This approach has gained attention in the development of alternative meats, mycofabrication of biocomposites, and production of mycelial leather and fabrics. These applications aim to replace animal husbandry and recycle agricultural waste for use in construction and electrical materials. This paper reviews current research trends on industrial applications of mushroom mycelia, covering strain improvements and molecular breeding as well as mycelial products and the production processes. Key findings, practical considerations, and valorization are also discussed.

**Graphical Abstract:**

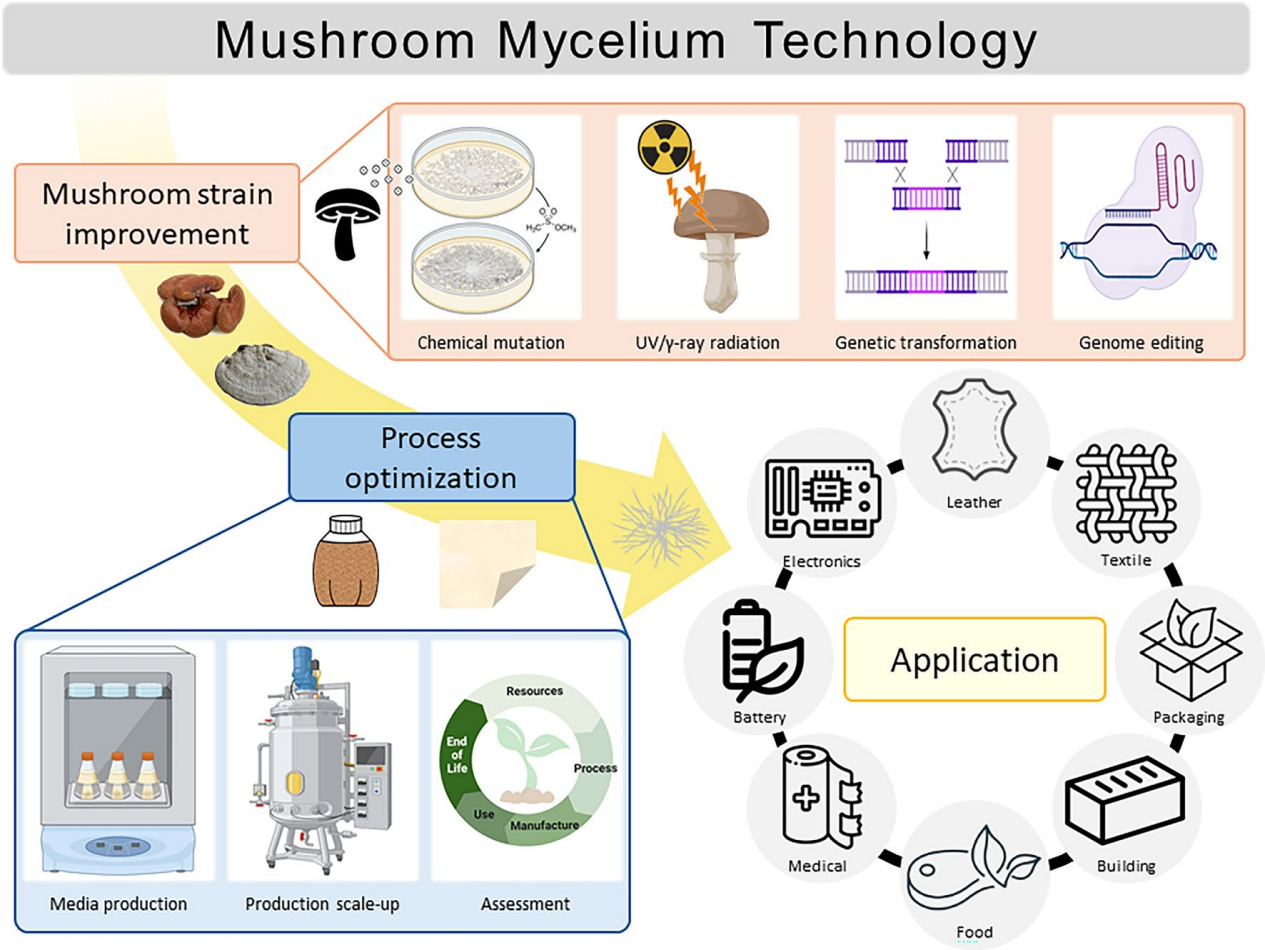

## Introduction

Recently, worldwide awareness of animal welfare and environmental protection has been increasing, and interest in vegan alternatives and decreased plastic use is rising. In particular, there is a movement to substitute plant materials for leather and meat, mimicking animal materials (Rollin 2006). Animal husbandry is responsible for 15% of total greenhouse gas emissions devastating land and water environments. The generation of odors and wastewater during the manufacturing process of meats and leathers is one of the leading causes of environmental pollution (Covington and Wise 2020). Accordingly, research on alternative materials and eco-friendly processes using renewable biomass is actively being conducted to reduce environmental pollution and pursue a healthy lifestyle (Lazar et al. 2023). Most industrially fabricated materials, such as construction and packaging materials nowadays, are nonrecyclable and environmentally unfriendly. Utilizing these conventional materials consumes energy, limits natural resources, and pollutes air, soil, and water bodies during production, transportation, and demolition. Eight to ten percent of global carbon dioxide emissions are released from manufacturing construction materials, and plastic bans have been registered in many countries (Rathore et al. 2021).

Fungi belong to a major group of eukaryotic organisms. They have a long evolutionary history since their emergence about 1.2–1.5 billion years ago (Wang et al. 1999). Among various fungal groups, saprotrophic fungi belonging to the Agaricomycetes class are attributed to the decline of terrestrial coal deposition at the early Permian period (300 million years ago) (Eastwood 2014; Hawksworth 2012; Floudas et al. 2012), although tectonic activities and climate conditions may also contribute to the carbon burial (Nelson et al. 2016). Among these, 155,603 species have been documented, which represents less than 10% of the estimated 2.5 million fungal species (Niskanen et al. 2023). Many fungal members have been used in the traditional biotechnology sectors, such as food fermentation, antibiotics, and industrial enzyme production. Some saprotrophic fungi are cultivated as major food crops, such as fruiting bodies of various mushrooms. 'Mushroom’ refers to fungal species that can produce large-sized fruiting bodies that are observable to the naked eye, including the mycelial network and fruiting body itself. Among mushroom-forming fungi, wood-decaying fungi have long been a subject of fascination for researchers, with their potential applications ranging from culinary delights to remediation of environmental pollutants. Hereafter, vegetative mycelia and fruiting bodies of wood-decaying Agaricomycete fungi are the focus of interest for environmentally friendly and sustainable materials for the future. In recent years, a dramatic increase in global mushroom fruiting body production has been driven partly by the growing awareness of their nutritional and therapeutic benefits (Bhagarathi et al. 2023). More recently, new trends have been shifting away from traditional biotechnology and applying fungal mycelia as eco-friendly resources in the formulation of alternative meats, fabrication of bio-composites, and production of mycelial leather, aiming to replace animal husbandry and recycle agricultural wastes (Madusanka et al. 2024; Aiduang et al. 2024; Yang et al. 2021; Antinori et al. 2020; Kim et al. 2017; Holt et al. 2012).

Mycelium is a fast-growing vegetative section of a fungus that is commonly found in biological and agricultural wastes. It is a harmless and sustainable material that bonds with the media to which it is attached. The Material Innovation Initiative, a non-profit think tank, has classified non-animal-based materials, excluding animal leather, fur, wool, down, and silk, as next-generation materials (materialinnovation.org 2022). To be classified as a next-generation material, an item must have a low negative environmental impact throughout its life cycle, including extraction, production, and consumption. Examples of next-generation materials include plant materials, fungal mycelia, cell cultures, microbes, and recycled materials. Based on the above definition, mushroom (mycelium) derived novel materials are next generation eco-friendly materials. Mycelium-based vegan leather and packaging are created by growing mycelia into customized growth chambers and molds, so-called mycofabrication (Raman et al. 2022). The field of mycelium-based leather and packaging has been expanding (Ariyani et al. 2024; Kniep et al. 2024; Madusanka et al. 2024; Amobonye et al. 2023; Raman et al. 2022). Patents in this area including various manufacturing methods are also abundant and publicly available (Elsacker et al. 2023). Beyond vegan leather/textile and packaging, mushroom mycelium-based materials expand into construction, electrical, and industrial materials (Ferrand 2024; Alaneme et al. 2023; Danninger et al. 2022). Although many review articles and research papers have been published on mycelium-based materials, there has been no review of the vertical approach from mushroom strains and mutation methods as biological resources to the mycelium production process. Therefore, this review discusses comprehensive studies from upstream (strain selection and breeding) to downstream (product development and process design) processing. Readers of this paper will have a more in-depth understanding of recent research on mushroom mycelium research and its limitations.

### Fungi and mushrooms as bioresources

#### Wood-decay fungi

Fungi, with their ancestral roots in aquatic habitats featuring flagellated spores, exhibit a closer evolutionary affinity to animals than plants. (James et al. 2006). Nonetheless, most of them have diversified by interacting with plants throughout evolution, colonizing terrestrial environments. They coexist with plants, decompose dead plant matter, or live parasitically on plants. There are symbiotic mushrooms like mycorrhiza that cannot degrade wood. Only certain Agaricomycetes are able to degrade wood efficiently, called wood-decaying fungi. Based on their decomposition abilities, they are roughly classified as white-rot fungi and brown-rot fungi.

Brown rot fungi can decompose cellulose but cannot fully break down lignin. They produce hydroxyl radicals generated from the redox reaction between H_2_O_2_ and Fe^3+^ ions through the Fenton reaction, oxidizing the crystalline cellulose and lignin in wood and creating cracks in the wood structure (Arantes and Goodell 2014). As the hyphae grow through these cracks, they secrete hemicellulase and endoglucanase enzymes to further decompose wood components such as hemicellulose and cellulose (Arantes and Goodell 2014; Krah et al. 2018). Consequently, wood degradation by these fungi leaves a brownish cubical fragmentation pattern on the wood. Fungal species such as *Coniophora puteana*, *Fomitopsis pinicola*, *Gloeophyllum trabeum*, *Postia placenta*, *Serpula lacrymans*, and *Wolfiporia cocos*, are some of the known brown rots (Floudas et al. 2012).

White-rot fungi are saprotrophs capable of completely decomposing lignin through the activity of oxidative enzymes, such as laccase and lignin-modifying peroxidases (PODs), leaving behind white, cellulosic, and hemicellulosic polymers in decaying wood (Cui et al. 2021). These polymers are further decomposed by cellulase and hemicellulase. White-rot fungi are the primary degraders in forests, and many species among them, such as *Agaricus bisporus*, *Lentinula edodes*, *Pleurotus ostreatus*,* Pl. eryngii*, and *Flammulina velutipes,* are consumed as edible mushroom. White-rot fungi generally possess multiple copies of genes for laccase, PODs, cellulase complex, and glucoside hydrolases (GHs) through paralogous gene expansion (Floudas et al. 2012). The following species are some of the known white-rot fungi: *Auricularia delicata*, *Fomitiporia mediterranea*, *Ganoderma lucidum, Heterobasidion annosum*, *Perenniporia fraxinea*, *Pl. ostreatus, Punctularia strigosozonata*, *Schizophyllum commune*, *Stereum hirsutum*, and* Trametes versicolor.*

Polyporales are saprotrophic or pathogenic mushroom species that produce shelf- or bracket-shaped fruiting bodies around tree trunks or branches. These fungi create strong mycelia as they attach to wood and produce sturdy, woody fruiting bodies. Bae et al. (2021) observed mycelial growth by cultivating 64 strains of Polyporales fungi, including *Tr. versicolor*, *Pycnoporus coccineus*, *Ga. lucidum*, and *Pe. fraxinea*, on sawdust and liquid media. Their findings revealed significant variation in growth rates at the strain level, with *Pe. fraxinea* exhibiting the fastest growth. Scanning electron microscope (SEM) analysis of the mycelial mat suggested that even among Polyporales strains, there were notable differences in both mycelial density and hyphal diameter, which may correlate with the strength of mycelial mat, demonstrating the necessity for further exploration and isolation of diverse strains.

### Fungal cell wall

Fungal mycelia is made of rigid tubes or cylinders of connected cells that grow on the surface of and inside solid substrates and produce aerial hyphae, forming a three-dimensional mycelial network. The three-dimensional structure of mycelium is a complex and interconnected network of fungal filaments (Islam et al. 2017). Mycelial strength is mainly attributed to cell wall polysaccharides (Gow et al. 2017). The fungal cell walls in higher fungi (fungi belonging to Ascomycota and Basidiomycota) consist of 10–20% chitin, 50–60% glucans, and 20–30% glycoproteins (Feofilova 2010; Bowman and Free 2006).

**Fig. 1:**
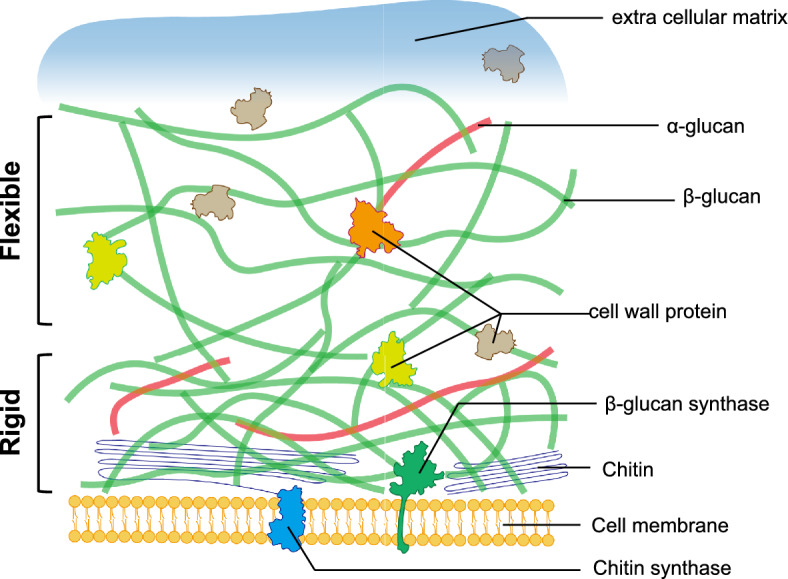
Fungal cell wall structure and its morphology. Cell wall is mainly composed of β-glucan, chitin, and proteins. The model is based on recent cell wall analysis data from *Sc. commune*

The cell wall components of mushrooms play a crucial role in determining their material properties and electrical characteristics. Comparing these aspects can provide insights into the significance of genetic modifications affecting mushroom cell walls (Fig. 1).

*Chitin* A long-chain polymer of N-acetylglucosamine that provides mechanical strength and rigidity to the cell wall (Ehren et al. 2020).

*β-glucans* Polysaccharides that contribute to the overall structure and flexibility of the cell wall (Ehren et al. 2020).

*Proteins* Present in the outer layer of the cell wall, proteins like mannoproteins and hydrophobins contribute to cell wall integrity and hydrophobicity (Haneef et al. 2017).

The cell wall structure of the Agaricomycete *Sc. commune* has previously been analyzed (Ehren et al., 2020, Safeer et al. 2023). Agaricomycete cell walls are assumed to consist of rigid chitin-glucan complexes in the inner cell wall and a more flexible network of β-glucans, extracellular polymeric matrix (EPS) and other proteins in the outer cell wall (Fig. 1) (De Beeck et al. 2021, Ehren et al. 2020, Safeer et al. 2023). The inner cell wall is made of different forms of chitin from single stranded to highly branched, as well as β-(1,3), β-(1,3)-(1,6)-glucan, and α-(1,3)-glucan (Ehren et al. 2020).

Chitin is a homopolymer of *N*-acetylglucosamine linked through β-1,4 glycosidic bonds catalyzed by membrane-bound chitin synthases (CHSs). Fungi have multiple copies of *CHS* genes classified into seven classes of CHS, belonging to two structurally distinct families (Roncero 2002; Lenardon et al. 2010). The number of CHS genes generally correlates with the cell wall chitin content (Latgé and Calderone 2006). Disruption of *CHS*s in *Aspergillus fumigatus* causes increased sensitivity to cell wall perturbing agents (Muszkieta et al. 2014). Deletion of *CSMA* or *CSMB*, belonging to Family II *CHS*, shortens chitin fibrils resulting in swollen conidia and reduced virulence (Muszkieta et al. 2014). In pathogenic yeast *Candida albicans*, the *chs8* mutant showed a defect in the synthesis of long-chitin microfibrils, which were found at the bud scar and septa, whereas the *chs3* mutant exhibited a defect in the accumulation of short-chitin rodlets as a cell wall component (Lenardon et al. 2007; Gow and Lenardon 2023). In the agaricomycete *Pl. ostreatus* disruption of basidiomycete-specific chitin synthases *chsb2, chsb3* and *chsb4* causes increased sensitivity to cell wall perturbing agents, thinner cell walls and decreased aerial hyphae production (Schiphof et al. 2024).

The glucan components are polymers of glucose units linked through β-1,3, β-1,6, α-1,3, and α-1,4 glycosidic bonds, with β-1,3-glucan being the main cell wall constituent (Beauvais et al. 2001; Papaspyridi et al. 2018; Liu et al. 2022a, b; Bowman & Free 2006). The synthesis of β-1,3-glucan is catalyzed by the membrane-integrated glucan synthase, Fks (Hu et al. 2023). Agaricomycete have fewer diverse *fks* genes than *chs* genes as they have 2–4 *fks* and 8–9 *chs* copies (Nakazawa et al. 2024; Grigoriev et al. 2014). Cell wall proteins are linked to chitin or glucans through charged amino acids (Safeer et al. 2023). EPS is a loosely packed matrix of mucilage that covers the cell wall. It comprises polymeric materials like polysaccharides and glycoproteins (De Beeck et al. 2021; Gow et al. 2017).

### Hyphae type

**Fig. 2:**
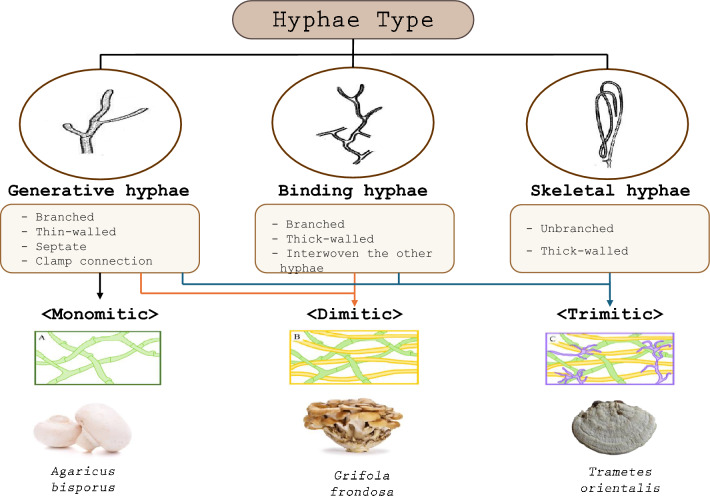
Classification of hyphae type and its mushroom example

As the mycelium is made up of hyphae, mycelium physiochemical properties of the mycelium depend on those of the hyphae. Therefore, identifying the shape and characteristics of the mycelium is one of the most important factors in the selection of mushrooms as a biological resource (Alaneme et al. 2023; Nadeem and Pirzada 2018; Montes et al. 2003). The terms monomitic**,** dimitic, and trimitic are used in mycology to classify fungi based on the types of hyphae present in their fruiting bodies and mycelia (Jones et al. 2020; Porter and Naleway 2022). These terms, in turn, relate to the functions and structural characteristics of three main types of hyphae: generative**,** binding, and skeletal hyphae (Fig. 2). Monomitic hyphae consist of exclusively generative hyphae, and are often saprotrophic, growing in softer environments like soil or decaying organic matter (e.g., *Ag. bisporus*). Dimitic hyphae are made of either generative and skeletal, or generative and binding hyphae (e.g., *Grifola frondosa*), whereas trimitic hyphae consist of all three types: generative, skeletal, and binding hyphae (e.g., *Tr. versicolor*). Dimitic and trimitic fungi are more specialized for lignocellulosic substrates like wood and often exhibit higher resistance to environmental stress, making them ecologically important in forest ecosystems and valuable for applications such as biodegradation and bioremediation. Nowadays, many mycelium-oriented companies use *Ganoderma* species for their products because of their trimitic hyphae structure (Porter and Naleway 2022).

### Mushroom strain improvement

#### Purpose of strain improvements

Various fungal species including Polyporales, *Fomes fomentarius, Pl. eryngii, Tr. versicolor*, and *Agaricus* have been explored for their potential in creating sustainable leather alternatives (Kniep et al. 2024). To improve the fundamental properties of mycelium-based materials, mycelium composites and/or mycelial mats with novel functions and characteristics can be developed by molecular breeding of fungi. With a shorter life cycle, mushroom-forming fungi have great potential to be developed as a new cultivar with a wider variety of properties compared to other material crops and farm animals. Many technologies such as chemical, physical, and molecular biological protocols have been introduced and successfully developed in some mushroom-forming fungi. This facilitates faster and easier molecular breeding to develop desired properties for future requirements such as food, medicine and sustainable biomaterials (Salazar-Cerezo et al. 2023; Dong et al. 2022; Lee et al. 2011; Sathesh-Prabu and Lee 2011). Moreover, their relatively small genome size makes it easier to check the whole genome sequence of isolated strains, which may ensure foreign-DNA-free edited cultivars are not classified as genetically modified organisms (GMOs) (Nakazawa et al. 2024). In the following sections, these breeding strategies will be introduced and discussed in more detail.

Generally, chemical and physical treatments were used to introduce random mutations and isolate a mutant strain with a suitable phenotype for breeding. In genetic breeding, a mating cross of two different strains is used to select favored progenies with desired phenotypes. In contrast, molecular breeding methods directly introduce a mutation in a targeted gene, or heterogeneous recombinant gene, in the cell, using genetic transformation and/or genome editing.

### Chemical treatments

The use of chemical reagents to mutate mushroom strains is an established technique in mycelium research, aimed at improving mushroom breeding and production characteristics. Chemical mutagenesis involves the use of chemical agents to induce mutations in the genetic material of organisms, leading to the development of new strains with potentially desirable traits. Chemical mutagens such as methanesulfonate methylester, an alkylating agent, have been used to treat basidiospores of mushrooms like *Hypsizygus marmoreus*. This treatment can yield mutant monokaryotic mycelia, which are then selected and mated to produce dikaryons with improved characteristics. For instance, some mutant strains have shown increased fruiting body production, while others exhibited unique morphological features such as flattened stipes and pilei (Lee et al. 2011). Chemical mutagenesis can lead to the development of strains that exceed parental cultivation characteristics, making them valuable for both commercial cultivation and molecular genetic studies. This method is particularly useful for generating resources to advance genetic research and deepen our understanding of the underlying mechanisms behind mutation-induced traits.

In the case of *Ganoderma lingzhi*, chemical mutagens like lithium chloride have been used to enhance the production of bioactive compounds such as polysaccharides and triterpenoids. This approach resulted in significant increases in yield compared to the original strains, demonstrating the potential of chemical mutagenesis in improving the commercial viability of mushroom strains (Ma et al. 2018). After inducing mutations, careful screening and selection of the desired mutant strains is crucial. This process often involves assessing morphological changes and growth characteristics to identify strains with beneficial traits. One of the advantages of using chemical mutagenesis is that the resulting strains are typically classified as non-transgenic. This classification can be beneficial in markets where non-genetically modified organisms are preferred. Overall, chemical mutagenesis is a powerful tool in mushroom strain development, offering the potential to enhance yield, improve growth characteristics, and increase the production of valuable bioactive compounds.

### Ultraviolet (UV) and gamma (γ) ray irradiation

Ultraviolet (UV) irradiation is widely used to induce DNA mutation in mushrooms due to its effectiveness in generating genetic diversity and enhancing desirable traits (Sathesh-Prabu and Lee 2011). UV light causes DNA damage, leading to mutations which can result in new phenotypes (Dong et al 2022). This method has been successfully applied to various mushroom species to improve traits such as yield, tolerance to environmental stress, and biosynthetic capabilities (Sun et al. 2022a, b; Harfi et al. 2021; Wang et al. 2014). UV irradiation has not yet been applied directly to mycelia for the purpose of developing mycelium-based materials. The effect of UV treatment on multinucleate hyphae remains an area worthy of further study. In particular, gamma irradiation has been suggested as a useful alternative to overcome the limitations of hybridization and the environmental and food safety concerns of genetically modified crops (Riviello-Flores et al. 2022). It can mutate fungal growth stages and mycelium properties to select targeted or useful variants, and is rapid and stable compared to other breeding methods (Sathesh-Prabu and Lee 2011). By irradiating monokaryon spores with gamma rays at appropriate doses ranging from 50 to 200 Gy, researchers have successfully induced mutations that result in diverse dikaryon mycelia (Kim and Yu 2014). Additionally, the analysis of genetic variation in different mushroom strains, such as *Hy. marmoreus* and *Lyophyllum* species, using techniques like random amplification of polymorphic DNA (RAPD) has demonstrated the effectiveness of gamma-ray irradiation in developing new mushroom strains with enhanced traits (Kim and Yu 2014). These findings highlight gamma irradiation as a crucial strategy for mushroom mutation, enabling the breeding of novel mushroom varieties with improved characteristics. Some mutant strains, including *Le. edodes, Ga. lucidium*, and *Sc. commune* showed good hyphae growth rate and density on saw-dust media (Kim et al. 2020). In these experiments, a number of variables need to be determined, including the effect of gamma-irradiation on genes and the propensity of the mutant strain to revert to the original parent strain.

### Genetic transformation

A conventional genetic transformation system is a core technology for introducing recombinant DNA or proteins into living cells and establishing new strains with desired phenotypes based on the introduced genetic factors. Two approaches have been used majorly to introduce recombinant DNA into the fungal cell: one depends on protoplast, and the other on a special kind of bacterium, *Agrobacterium tumefaciens. Ag. tumefaciens*-mediated transformation (ATMT) method has an advantage in that it does not require a protocol for efficient protoplast production (de Groot et al. 1998; Kim et al. 2015). However, the ATMT method does not yield large numbers of transformants in a single experiment, and the introduced DNA almost always integrates on the chromosome, classifying resulting strains as GMOs. Furthermore, the ATMT method cannot directly introduce proteins into the cell.

Gene targeting technology can be used to disrupt or introduce a desired change in a gene of interest. When a gene is disrupted in this manner it is referred to as a gene knock-out However, developing gene-targeting technology is challenging because the homologous recombination system is active only during meiotic division just before basidiospore formation and insufficiently active in the vegetative mycelia or during isolation of transformants from protoplasts. Non-homologous end joining (NHEJ) is the other repair system, predominant through most of the fungal life cycle. In a limited number of model species such as *Coprinopsis cinerea* (Nakazawa et al. 2011)*, Sc. commune* (Jan Vonk et al. 2019) and *Pl. ostreatus* (Salame et al. 2012), specific genes required for NHEJ system were disrupted to develop a strain with an efficient homologous recombination system.

Conventional genetic transformation methods typically involve the presence of foreign DNA sequences, due to the use of a genetic marker gene for the screening of transformants. In most cases, genetic markers contain recombinant DNA composed of multiple origins of organisms (Matsunaga et al. 2017). Limited numbers of genetic markers are known as a ‘self-cloning marker’, such as *pyrG* (Nakazawa et al. 2016) or *Cbx*^R^ markers (Honda et al. 2000). It must be considered which kind of markers or what kind of purposes the conventional transformation must be considered when developing new strains aimed at future development of mycelial composites or food productions.

### Genome editing

Genome editing is a promising technology for molecular breeding in many organisms including mushroom-forming fungi. The most popular and efficient genome editing technique, CRISPR/Cas9 (Jinek et al. 2012; Song et al. 2019) was successfully introduced into various mushroom-forming fungi including *Cop. cinerea* (Sugano et al. 2017), *Sc. commune* (Jan Vonk et al. 2019), *Pl. ostreatus* (Boontawon et al. 2021b; Xu et al. 2022; Yamasaki et al. 2022; Koshi et al. 2022), *Le. edodes* (Moon et al. 2021; Kamiya et al. 2023), *Ga. lucidum* (Qin et al. 2017; Liu et al. 2020), *Ag. bisporus* (Choi et al. 2023), *Pl. eryngii* (Wang et al. 2021), *Fl. filiformis* (Liu et al. 2022a, b), and *Gelatoporia* (formally, *Ceriporiopsis) subvermispora* (Nakazawa et al. 2022). CRISPR/Cas9 requires two essential components: a Cas9 protein that introduces DNA double-strand break (DSB) and a guide RNA (gRNA) that recruits Cas9/gRNA complex to the target site on the chromosome through base pair formation with its corresponding single stranded DNA. Generally, the introduced DSB is repaired mainly by the NHEJ repair system in the cell. However, small insertions or deletions of base pairs may happen at a certain frequency during the repair process. In a case where no mutation is introduced, the target site would be attacked repeatedly by the Cas9/gRNA complex until a mutation occurs. In this way, when using a gRNA sequence specific to the target site, CRISPR/Cas9 can be used to disrupt a gene of interest (Boontawon et al. 2021a; Nakazawa et al. 2023). With the co-transformation donor DNA consisting of DNA fragments flanked by homologous arm sequences (HR arms) to a target gene, CRISPR/Cas9 can efficiently catalyze the exchange of DNA sequences between the chromosome and donor DNA through homology-direct repair (HDR) at the target site (Jan Vonk et al. 2019). The efficiency of gene editing with CRISPR/Cas9 combined with HR arms is generally greater than with CRISPR/Cas9 alone; however, the effectiveness can vary depending on the length of the HR arms and the fungal species involved (Zhang et al. 2016; Fang et al. 2016; Pohl et al. 2016). In this way, a desired mutation can theoretically be designed and introduced into any gene of interest.

For material use, genetically modified (GM) mushroom-forming fungi are more acceptable compared to edible consumption. In this regard, conventional genetic modification, as well as genome editing, can be powerful tools for molecular breeding. The genes to be disrupted, modified or overexpressed should have meaningful traits to be molecular breeding targets. Identifying the genes responsible for cell wall synthesis, growth, and aerial hypha formation, is indispensable for elucidating cell wall formation in mushroom-forming fungi and for developing new materials with enhanced properties not found in current mycelial materials derived from natural strains.

### Possible target genes for molecular breeding

The cell wall is an ideal target for improving the physical properties of mushroom materials, as it is closely related to the stiffness or smoothness of the mycelium mat, as discussed in the mushroom composites and packaging section. To dynamically change the cell wall structure, transcriptional regulatory systems of cell wall synthesis genes are potential targets. In filamentous fungi, the cell wall integrity (CWI) signaling pathway is known to be an important transcriptional regulatory system for cell wall synthesis (Yoshimi et al. 2022). The canonical CWI pathway is partially conserved in different fungal species. It typically comprises cell wall sensors at the cell surface, guanine nucleotide exchange factors (GEFs), Rho GTPases, protein kinase C (PKC) and a mitogen-activated protein kinase (MPK) module (Dichtl et al. 2016).

In basidiomycetes, the CWI signaling pathway in *Cryptococcus neoformans* has been well studied. In *Cr. neoformans*, the major components of the CWI signaling pathway were conserved, with the exception of the membrane-anchored sensor proteins (Dichtl et al. 2016). In the yeast-like basidiomycetes *Ustilago maydis* and *Cr. neoformans*, Rho1 is essential for growth (Dichtl et al. 2016). In *U. maydis*, the necessity of Rho1 for cell division and chitin deposition was demonstrated using a strain where Rho1 driven by the *crg1* promoter (Pham et al. 2009). However, in *Ga. lucidum*, downregulation of *slt2*/*mpk1* by RNAi resulted in a moderately strong growth defect on both agar and substrate medium (Zhang et al. 2017). In this strain, both the amount of glucan and chitin in the cell wall and the expression of the corresponding biosynthetic genes were reduced. Thus, in basidiomycetes, MPK1 has a strong effect on growth while allowing modification of cell wall composition, suggesting that downstream transcription factors are promising breeding targets. Indeed, in *Ga. lucidum*, downregulation of the *swi6* transcription factor by RNAi leads to a decrease in β-glucan and chitin with minor growth defects (Lian et al. 2021).

### Mycelium production process and mycelium-based products

#### Classification of mycelium-based products

**Fig. 3:**
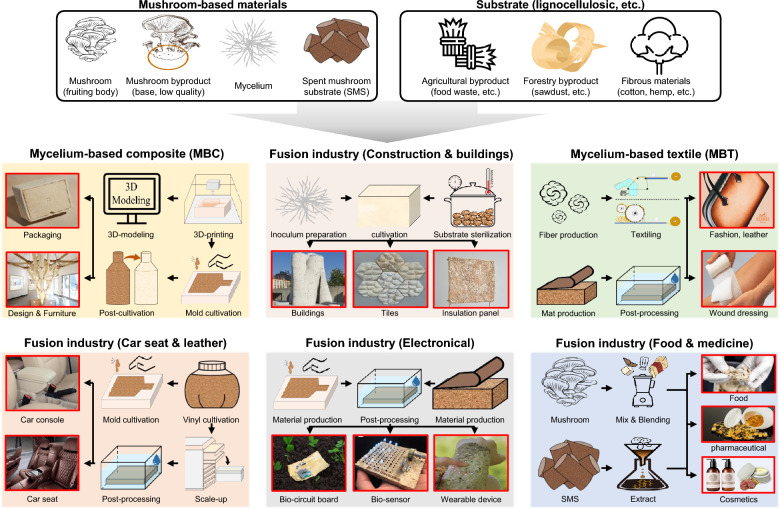
Schematic of the production of mycelium-based materials using mushroom mycelium and industrial substrates

Mushroom mycelium has emerged as a versatile and sustainable material with applications across several industries. These applications include mycelium-based composite, mycelium-based textiles, and many fusion technologies (Fig. 3). Commercialization has been successful in some product areas (textiles), and many efforts are underway to commercialize in the areas of high-performance materials (composites and fusion products) and mass production (leathers and alternative meats). Innovative applications of mushroom mycelium face several significant challenges that hinder large-scale commercialization. A more detailed breakdown by product is as follows.

*Packaging* Mycelium-based composite materials are being developed as biodegradable alternatives to plastic and foam packaging. These decompose in as little as 30 days and release nutrients into the soil, making them an eco-friendly option for reducing packaging waste (Alaneme et al. 2023; Enarevba and Haapala et al. 2023; Abhijith et al. 2018). If the challenges of growth and production (slow growth rate and scaling limitations) are sufficiently addressed, we expect to see a wider range of products coming to the market soon.

*Construction and building materials* Mycelium composites are being researched for use in construction as insulation panels, building panels, bricks, and sound panels. Their low density and water resistance properties make them promising alternatives in the building industry (Alaneme et al. 2023; Gou et al. 2021; Joshi et al. 2020; Jones et al. 2020). However, the irregular growth patterns of mycelium lead to non-uniform thickness and unpredictable properties, making it difficult to achieve standardized products.

*Textiles and fashion* Mycelium is being used to create leather alternatives for the fashion industry. Mycelium-based leather can be dyed and bleached like animal leather but has a shorter production time of about 5 days compared to 6–8 weeks for animal leather. It is also biodegradable and addresses ethical concerns related to animal farming (Crawford et al. 2024; Silverman et al. 2020; Bustillos et al. 2020; Cerimi et al. 2019). However, mycelium-based leathers have relatively low mechanical strength, moisture sensitivity and limited thickness, affecting commercial applications such as car seats.

*Food industry* Mycelium is being used to develop meat alternatives and protein sources. Mycoprotein, derived from the mycelia of *Fusarium venenatum*, has been used in plant-based foods since the 1960s. These fungi-based foods produce 40–52% less pollution compared to livestock farming (Amara and El-Baky 2023; Souza Filho et al. 2019). Mycelium-based food ingredients must compete with traditional food ingredients that have established supply chains and familiar properties.

*Biomedical applications* Fungi are being researched for their potential in synthesizing biomaterials with applications in medicine and pharmacology (Manan et al. 2021). The lack of a standardized approach to production methods or material characterization makes it challenging to ensure consistent quality. In addition, the novel nature of mycelium-based materials may lead to unclear or complex regulatory pathways for certain applications.

*Environmental remediation* Mycelium is being explored for its potential in bioremediation and environmental cleanup applications (Akpasi et al. 2023). Commercialization requires field studies in a wider range of areas. Additionally, issues related to intellectual property must be addressed. A few companies hold essential patents, restricting knowledge distribution and hindering widespread production.

*Automotive industry*: pressed mycomaterials are being researched for potential use in car parts (Jones et al. 2021). Mycelium-based materials have relatively weak structural properties, limiting their use in load-bearing applications. Mycelium-based products must compete with traditional materials including plastics and fiber-reinforced plastics (FRP).

Design and furniture: mycelium is being used to create various design objects and furniture pieces, such as lamps and chairs (Alemu et al. 2022; Yang et al. 2021). These small indoor suites are being commercialized in a variety of areas, including designer products. One issue is that the unique appearance and texture of mycelium materials may not align with traditional consumer preferences.

Electronics and batteries: the unique properties of living mycelium networks allow for the propagation of electrical signals, suggesting potential applications in analog electronics and unconventional computing (Mayne et al. 2023). The integration of mycelium with cellulose-based materials further enhances the development of biodegradable batteries, capable of generating power densities suitable for small electronic devices (Danninger et al. 2022). Along with uniform quality control issues, growing large volumes of mycelium present logistical difficulties, especially for on-site production.

These diverse applications showcase the potential of mushroom mycelium as a sustainable, biodegradable, and versatile material across multiple industries, offering eco-friendly alternatives to traditional materials. Overcoming these challenges mentioned above will require continued research, development of standardized production methods, and efforts to educate both industry professionals and the public about the potential of mycelium-based materials. Collaboration between academia, industry, and regulatory bodies will be crucial in addressing these obstacles and unlocking the full potential of mushroom mycelium in various applications.

Many mycelium-based products (from packaging to electronics) are produced using the solid-state culture process, as shown in Fig. 3. Before the main production stage, liquid culture and small-scale solid-state culture must be performed to make inoculum and subculture materials. To describe the process more comprehensively, substrate and culture media must first be dealt with.

In the case of mycelium-based composite (MBC) and car console production, 3D modeling is essential to creating the formwork for packaging and designing furniture. Some treatments to prevent fire and external contamination are important for construction and building applications. Mycelium-based textiles (MBT) and leather require the application of chemical processes such as tanning, dyeing, and surface coating used in conventional leather processes. For electronic applications, this is complemented by cultivation techniques to fine-tune the shape and density of the hyphae, as well as post-processing techniques depending on the application. Developing food, cosmetic, and pharmaceutical ingredients using mycelium requires effectively utilizing edible strains and spent mushroom substrate (SMS).

### Culture media and solid substrates

Cultivating mushroom mycelium efficiently requires careful consideration of the culture media and solid substrates employed. Mushroom growers have long recognized the importance of optimizing culture conditions to enhance mycelial growth and productivity. One study focused on the entomogenous fungus *Aschersonia aleyrodis* highlighted the significance of optimizing nutritional requirements for both mycelial growth and sporulation. (Zhu et al. 2008). When the mycelium of a mushroom creates a three-dimensional structure and this structure grows in length, it creates a layer of mycelium of a certain thickness, called a mycelium mat. These mats can be used as raw materials for mushroom leather and mushroom pulp, and when combined with other biomass, they form mycelium composites. Culture substrates are sterilized and cooled in a vinyl bag, inoculated with mushroom hyphae, cultured for a certain period of time, and then crushed and put in a container for mycelium mat production and secondary culturing (Gandia et al. 2022; Raman et al. 2022). However, this method has disadvantages as it is time-consuming due to the need for two rounds of culturing and carries a high contamination risk. In addition, establishing optimal culture conditions has been attempted using a more effective medium than potato dextrose broth (PDB), which is a medium commonly used for culturing mushroom mycelium (Haneef et al. 2017). This study aimed to find an efficient method to shorten mycelium mat production time, reduce contamination risk, and improve mycelium mat quality, thereby increasing productivity and economic value. Culture media composition plays a crucial role in supporting the growth and metabolism of mushroom mycelium. Techniques such as orthogonal design, response surface design, and uniform design, have demonstrated effectiveness in optimizing fermentation media, thereby enhancing fermentation levels efficiency and enzyme production. This approach can be extended to optimizing culture media for mushroom mycelium cultivation by exploring the optimal concentrations of various nutrients and carbon sources. Recently, a paper was published on the importance of disaccharides as a carbon source in mushroom mycelium cultures (Nussbaum et al. 2023). Malt concentration in solid substrate influences mycelial growth and network connectivity in *Ganoderma* species, impacting radial growth kinetics, mycelium density, and mechanical characteristics like Young's modulus. In addition, the effect of agar, another carbohydrate component, is also being studied. In the past, agar was not considered an energy or carbon source, but it has recently been recognized as an important factor in the growth of hyphae and mycelium. When the agar concentration was increased from 1.5% to 6.0% agar, the mycelium density and hyphal width increased by 32.3% and 63.6%, respectively. The implications of these findings will allow for the advancement and tuning of fungi-based materials, particularly for the application of sustainable textiles and fine particulate filters (Hotz et al. 2023). In the future, the effects of different carbohydrate components, including maltose and agar, should be studied more closely. Table 1 summarizes media for strains stock, liquid media for prepare mycelium inoculum and solid media for mycelium production.

**Table 1:** Summary of culture medium and solid substrate for the production of mushroom mycelium

Strain	Medium for strain stock	Liquid medium to prepare mycelium inoculum	Solid medium for mycelium mat production	Substrate for mycelium composite	References
*Abortiporus biennis*	Malt extract glucose agar	Low nutrient media	–	Wheat bran, Wheat straw, Coconut husk, Broadleaves sawdust	Balaes et al. (2023)
*Ceriporia lacerata*	Potato dextrose agar	Potato dextrose medium	–	Soybean straw	Shao et al. (2016)
*Fomitopsis pinicola*	Malt extract peptone agar	1% Sugar, 1% Malt, 0.2% Yeast	Woven fabrics, Woven felts	Woven fabrics, Woven felts	Kniep et al. (2024)
*Fomitella fraxinea*	Potato dextrose agar	Yeast malt peptone medium	Sawdust substrate	–	Raman et al. (2022)
	Potato dextrose agar	Potato dextrose medium	–	Sawdust substrate	Bae et al. (2021)
*Fomes fomentarius*	Malt extract agar	Malt extract medium	–	Hemp shives	Pohl et al. (2022)
	Malt extract glucose agar	Low nutrient media	–	Wheat bran, Wheat straw, Coconut husk, Broadleaves sawdust,	Balaeș et al. (2023)
*Ganoderma lucidum*	–	–	–	Bamboo fiber substrate	Soh et al. (2020)
	Potato dextrose agar	Potato dextrose medium	Microcrystalline cellulose	–	Haneef et al. (2017)
	–	–	–	Red oak sawdust, Cotton carpel	Lingam et al. (2023)
	–	–	–	Beech sawdust, cotton fiber, soy silk fiber, wheat bran	Vasatko et al. (2022)
	Grain mixture	Grain mixture	–	*P. eryngii* Spent mushroom substrate with wheat bran, wheat meal, oil seed	Schritt et al. (2021)
	Grain mixture	Hemp hurds + maple veneers	–	Hemp fibers, Hemp hurds Pine wood sawdust and shavings	Özdemir et al. (2022)
*Ga. resinaceum*	–	–	–	Hemp shives & Soybean hulls	Adamatzky and Gandia (2022)
	Dark Malt extract agar	Dark Malt extract medium	–	Wheat straw	Xing et al. (2018)
*Letinus sajor-caju*	Wood	Potato dextrose medium Sorghum grain	–	Corn husk, Wood sawdust, 5% Rice bran, 1% Calcium carbonate, 2% Calcium sulfate, 0.2% Sodium sulfate	Teeraphantuvat et al. (2024)
*Trametes multicolor*	–	–	–	Rapeseed straw, Beech sawdust	Appels et al. (2019)
*Tr. versicolor*	Potato dextrose agar	Potato dextrose medium	–	Sawdust substrate	Bae et al. (2021)
	Grain mixture	Grain mixture	Bacterial cellulose + hemp fibres	–	Elsacker et al. (2021)
	Malt extract agar	Malt extract agar + sterile corn steep liquor	–	*Betula alleghaniensis* Britt. wood veneers	Sun et al. (2022a, b)
	Malt extract glucose agar	Low nutrient media	–	Wheat bran, Wheat straw, Coconut husk, Broadleaves sawdust	Balaeș et al. (2023)
*Tr. orientalis*	Potato dextrose agar	Yeast malt medium	Sawdust substrate	–	Jeong et al. (2023)
*Pleurotus ostreatus*	–	–	–	Cotton	Appels et al. (2019)
	Potato dextrose medium	Potato dextrose broth	Microcrystalline cellulose	–	Haneef et al (2017)
	Potato dextrose agar	Potato dextrose broth + 1% cellulose	–	Sugar Cane, Sawdust, wheat bran,	
	–	–	–	Straw, Sawdust, Bagasse + sawdust + wheat bran	Lingam et al. (2023)
	–	Glucose agar	–	Rubber wood sawdust + corn grain	Shakir et al. (2020)
	–	–	Hemp bedding substrate	–	Adamatzky and Gandia (2021)
*Pl. sanguineus*	Potato dextrose agar	Potato dextrose medium	–	Coconut powder + wheat bran	Gou et al. (2021)
*Pl. eryngii*	–	–	–	Sawdust substrate	Silverman et al. (2020)
	Malt extract peptone agar	1% Sugar, 1% Malt, 0.2% Yeast	Woven fabrics Woven felts	Woven fabrics Woven felts	Kniep et al. (2024)

Solid substrates also present an important factor in the cultivation of mushroom mycelium. Using agricultural waste or byproducts as solid substrates offers a cost-effective and sustainable solution, while potentially influencing the growth characteristics and secondary metabolite production of the mycelium (Ángeles-Argáiz et al. 2020). Most agricultural residues rich in lignocellulose, such as wheat straw, rice straw, corn cobs, and sugar cane, serve as ideal substrates for fungal growth due to cellulose, hemicellulose, and lignin composition. These materials typically contain 30–50% cellulose, 20–30% hemicellulose, and 5 to 30% lignin. Predictions suggest that global waste production could reach 2.59 billion tons by 2030 (Awogbemi and Von Kallon 2022). Therefore, the efficient utilization of various cellulosic by-products generated globally as solid media for raw materials is expected to significantly contribute to carbon reduction and the economic production of mushroom mycelium. Representative substrates for mycelium composite production reported so far are shown in Table 1.

The optimization of culture media and solid substrates is crucial for enhancing the efficiency and productivity of mushroom mycelium cultivation. By drawing upon insights from related fields, such as mushroom research and fermentation medium optimization, researchers and growers can develop innovative strategies to unlock the full potential of mushroom mycelium production.

### Mushroom leather and pulp

Mushroom leather and pulp production using mycelium presents a promising avenue for sustainable material development. Mycelium-based leather, derived from various fungi, has shown mechanical properties comparable to traditional leather, particularly when reinforced with substrates like needle felts or woven fabrics, enhancing tensile strength beyond that of synthetic alternatives (Kniep et al. 2024; Raman et al. 2022). While various mushroom leathers have been researched and are available from some companies, they are not comparable to traditional leather in terms of texture or durability. Furthermore, there are still too many affordability hurdles to overcome. As a reflection of this, some mushroom leather companies in the US have ceased production (Chan and Webb 2023). Studies are actively being conducted to meet the industrial standard of leather and pulp through physical and chemical treatment processes (Appels et al. 2020; Raman et al. 2022).

Mushroom pulp, often referred to as mycelium pulp, is an innovative bio-based material derived from the mycelial structure of mushrooms. The production of mycelium pulp, achieved through innovative extraction methods from fruiting bodies, retains the structural integrity of the mycelium, resulting in a versatile material with excellent deformability suitable for diverse applications, including packaging materials, textiles, and even building materials (Nakauchi et al. 2023). Additionally, the cultivation of mycelium on paste substrates has been explored to improve nutrient availability and scalability, further supporting the feasibility of mycelium-based products (Crawford et al. 2024). However, challenges remain in replicating the full properties of leather and optimizing processing techniques, indicating a need for continued research in this field (Kniep et al. 2024; Raman et al. 2022). Mycelium offers a sustainable alternative to conventional materials, aligning with eco-friendly design principles.

### Mushroom composites and packaging (plastic alternatives)

Among the materials, the most widely used material is plastic. There are many types of plastic and it is the most commonly encountered material in our daily life. However, since it is a petroleum-based material, even if the oil depletion problem is solved, the environmental problem remains a big issue. Mushroom mycelium presents a promising alternative to traditional plastic packaging, leveraging its biodegradable and sustainable properties. Mycelium, derived from agricultural waste, forms robust, self-assembling materials that can replace polystyrene in packaging applications due to its environmental friendliness and ability to degrade naturally (Patel and Sharma 2023). Research highlights the versatility of mycelium composites, which exhibit high stability, flexibility, and mechanical strength, making them suitable for various uses, including insulation and packaging (Verma et al. 2023; Zhang et al. 2023). Innovative approaches, such as glycerol treatment, have enhanced the toughness and hydrophilicity of mycelium films, further improving their applicability in packaging (Amobonye et al. 2023). Additionally, studies on mycelium composites derived from hemp shives indicate complete biodegradability within 12 weeks, although challenges like high water absorption and mold resistance remain (Loris et al. 2022). While mycelium-based materials show great potential, ongoing research is necessary to optimize their properties for broader commercial use. To meet the industrial standards of plastic products including packaging, the development of lightweight materials with high stiffness is essential. In addition, research on optimizing production systems for commercializing mycelium-based materials is ongoing. Despite this high possibility, mass production using mushroom mycelium has not yet been reported in the paper but strategies for growing large-scale mycelium structures were suggested (Dessi-Olive 2022). Several companies continue to promote the mass production of mushroom composites (Bearne 2023).

### Alternative (artificial) meat

The representative of alternative livestock products is artificial meat. Artificial meat can be divided into two main methods: one involves mimicking the taste and texture of meat by processing plant materials, while the other involves culturing animal cells in specific areas and conditions. These artificial meats have some advantages over conventional meat in terms of safety and animal cruelty issues. Alternative meat production using mushroom mycelium presents a promising avenue for addressing global protein demands while mitigating environmental impacts. Research indicates that filamentous fungi, particularly mycoprotein derived from mycelium, can serve as nutritious meat substitutes, with studies showing high protein content (19.4% w/w) and beneficial fatty acid profiles when cultivated on food industry byproducts like tempeh residual water (Wikandari et al. 2023). Additionally, various fungal species, such as *Fusarium venenatum* and *Le. edodes*, have been identified as suitable candidates for meat replacement due to their nutritional profiles and rapid growth rates (Kok Kee et al. 2022). Moreover, edible mycelium can function as a support structure in cultivated meat production, enhancing cell proliferation and differentiation, which is crucial for developing meat-like textures (Ogawa et al. 2024). Despite challenges in mycelium productivity and sensory acceptance, advancements in culture medium optimization show potential for improving these aspects (Majumder et al. 2024). Overall, leveraging mushroom mycelium could significantly contribute to sustainable alternative protein sources (Holt et al. 2024).

### Construction materials

Mycelium composites are emerging as a promising sustainable alternative in the construction industry due to their eco-friendly properties and potential for innovation. These materials are derived from the vegetative part of fungi, known as mycelium, and can be combined with lignocellulosic materials to form mycelium-based composites (MBCs). These composites are characterized by their low density, elasticity, and cost-effectiveness, making them suitable for various applications in construction and other industries (Madusanka et al. 2024). Mycelium composites offer several advantages over traditional materials. They are biodegradable, contribute to zero waste during production, and can be tailored through genetic and biochemical modifications to enhance their properties. The versatility of MBCs allows them to be used in material-driven design (MDD) approaches, which has the potential to foster innovative applications in construction and other industries (Madusanka et al. 2024). Despite their potential, mycelium composites face several challenges. The production process can have a significant environmental impact, particularly in regions where fossil fuels are the primary energy source. For example, in South Africa, the high electricity consumption required for production can make mycelium composites less sustainable than conventional materials like concrete. Additionally, the shorter lifespan of these composites necessitates frequent replacements, further increasing their environmental footprint (Akromah et al. 2024).

Ongoing research is focused on overcoming these challenges by improving the biological characteristics of mycelium through bio-fabrication procedures and optimizing the production process (Alaneme et al. 2023). Researchers are also conducting life cycle assessments to understand the environmental impacts better and to develop strategies for reducing them, such as using alternative energy sources (Volk et al. 2024; Alaux et al. 2024; Enarevba and Haapala et al. 2023; Williams et al. 2022). Collaborative efforts in material science and fungal biotechnology are encouraged to address these issues and unlock the full potential of mycelium composites for sustainable construction. While mycelium composites hold great promise for sustainable construction, their environmental impact and production challenges must be carefully managed. Continued research and innovation are essential to enhance their viability as a green building material.

### Electronics

The use of mushroom mycelium in biocomputing and biosensing is an emerging field that explores the potential of exploring fungi as computing devices and sensors (Fukasawa et al. 2024; Phillips et al. 2024; Mayne et al. 2023; Danninger et al. 2022). Filamentous fungi, such as those found in mycelial networks, have long been recognized for their ecological and biotechnological importance, yet synthetic biologists have historically overlooked them for electronic application. The increased complexity of their genomes and the lack of molecular tools have slowed the pace of innovation in filamentous fungi engineering. However, recent advancements in synthetic biology have unlocked the potential of these remarkable organisms, paving the way for their integration into the world of biocomputing and biosensing (Jo et al. 2023). The mycelium is capable of nontrivial mapping of electrical signals due to the substrate's nonlinear electrical characteristics, which allows it to function as a living electronic component (Roberts and Adamatzky 2022). Mycelia, the branching, web-like root structure of fungi, can act as conductors and electronic components (Adamatzky and Gandia 2021). This capability is being investigated by researchers, such as those at the Unconventional Computing Laboratory, who are developing fungal computing (so called mushroom computers) and electronics by leveraging the action potential-like spikes produced by fungi, similar to neuronal spikes in the brain (Hu 2023). These spikes can be used to represent binary data (zeroes and ones) and to implement basic logical and electronic circuits (Adamatzky and Gandia 2021). Fungal mycelium has been shown to increase conductivity and communication speed when stimulated at two separate points, which allows for the establishment of memory, akin to how brain cells form habits. Different geometries of mycelium can compute various logical functions, which can be mapped based on its electrical responses (Phillips et al. 2024). Nowadays, taking advantage of fungal mycelia’s natural light sensitivity, Mishra et al. (2024) developed an electrical interface to both house the mycelia and measure their electrophysiological action potentials. The following are potential applications:

*Environmental sensors* Fungal networks can be used as large-scale environmental sensors. They can monitor data flows and provide insights into ecosystem changes by interpreting the electrical signals used by fungi to process information (Dehshibi et al. 2021).

*Biodegradable electronics*: fungal machines offer the potential for creating self-assembling, self-repairing, and biodegradable electronic devices. These could be used in situations where traditional electronics are not feasible or environmentally friendly (Danninger et al. 2022).

*Wearable technology* researchers are exploring the use of mycelium networks in wearable technology. Fungal skins have shown potential as touch sensors in robotics and can sense light, making them suitable for various wearable applications (Adamatzky et al. 2021).

*Structural and building materials* fungal mycelium can create structural substrates for buildings that self-grow, build, and repair themselves. These structures could include embedded sensorial elements for environmental monitoring (McGaw et al. 2022).

*Optimization and artificial intelligence*: mushroom computers can solve complex problems in fields like artificial intelligence and optimization by leveraging the adaptability and complexity of living fungal networks (Wainaina and Taherzadeh 2023).

These applications highlight the unique capabilities of fungal computing, particularly in areas where traditional computing technologies may face limitations. However, it is important to note that fungal computing is still in the research phase, and further development is needed to realize its potential in everyday devices. In addition, applications in this field highlight further beneficial mycelial properties, differing those of the aforementioned mycelium materials.

### Capacitors and batteries

Capacitors and batteries are essential components in modern electronic devices, serving different yet complementary functions. Capacitors are essential components in electronic circuits, playing a crucial role in energy storage, filtering, and signal coupling. In recent years, the development of electrochemical capacitors, also known as supercapacitors, has garnered significant attention due to their superior power capabilities and exceptional life cycle (Zhao and Burke 2021). One approach to enhancing supercapacitor performance is exploring new materials, such as fungal-derived compounds (Szacilowski et al. 2023). Recent trends in fungal capacitor research highlight the innovative use of mycelium and fungal-derived materials in energy storage applications. Studies indicate that mycelium, particularly from species like *Pl. ostreatus*, exhibits promising capacitive properties, with capacitance values in the hundreds of picofarads and a voltage-dependent pseudocapacitance reaching hundreds of microfarads. This suggests potential for mycelium as an organic alternative in capacitor technology, especially in dense arrays for charge storage (Szacilowski et al. 2023). Additionally, research on biomass-derived fungal carbons, such as those from *Ag. bisporus* and *Pl. eryngii*, revealed their high surface areas and specific capacitance, making them suitable for supercapacitor applications. These materials can be tuned for optimal performance through variations in growth conditions and species selection, achieving specific capacitance values exceeding those of traditional carbon materials (Jones et al. 2024). Furthermore, activated carbon derived from black *Aspergillus* demonstrates exceptional gravimetric capacitance and cycling stability, indicating a sustainable pathway for high-performance supercapacitors (Yang et al. 2021). Overall, the integration of fungal materials into capacitor technology represents a significant advancement in sustainable energy storage solutions.

The relationship between these two energy storage devices has become increasingly important, especially with the rise of emerging technologies such as lithium-ion batteries and their potential applications. (Coelho et al. 2023; Docimo et al. 2014; Porcarelli et al. 2012). Lithium-ion batteries are largely composed of a positive electrode, a negative electrode, a separator, and an electrolyte. A lithium-transition metal oxide-based compound is primarily used as the positive electrode material. Mushroom mycelium has been explored as a sustainable and flexible material for the fabrication of microsupercapacitors, which can serve as an alternative to traditional lithium-ion batteries (Coelho et al. 2023). These mushroom-based microsupercapacitors exhibit desirable characteristics such as high-power densities, long life cycles, and fast charge–discharge rates, making them a promising option for various portable and wearable technologies. Mushroom mycelium contributes to sustainable battery production through its application as a biodegradable substrate and as a source of bioactive materials. Research demonstrates that fungal mycelium skins can be processed into flexible electronic devices, exhibiting high conductivity and durability, essential for battery applications (Danninger et al. 2022). These mycelium-based batteries have approximately 3.8 mAh cm^−2^ capacities, effectively powering small electronic devices like sensors (Danninger et al. 2022). Additionally, mycelium residues from fermentation processes, such as those from *Aspergillus niger*, can be converted into biochar, which serves as an anode material for lithium-ion batteries. This approach utilizes waste and enhances battery performance due to the nitrogen and silicon content in the mycelium (Gu et al. 2023). Mushroom-derived biomasses and mycelia are being extensively studied as anode materials for batteries (Li et al. 2024; Xu et al. 2023; Han et al. 2020; Tang et al. 2016; Campbell et al. 2015). Research shows that the anode material of mushroom-based components can omit the activation process, and is environmentally friendly as the use of various anode activation and coating reagents. These findings collectively highlight mycelium's potential in advancing sustainable battery production while addressing environmental concerns associated with electronic waste.

### Mycelium-production bioprocess

In the last decade, great progress has been made in mushroom mycelium research, from basic research to various applied materials. However, for mushroom mycelium research to make significant industrial progress, large-scale production and corresponding bioprocessing systems are urgently needed. In particular, we are convinced that the large-scale production and utilization of mushroom mycelium will increase dramatically if even a partial continuous process is possible, as the current process is still batch-based.

Mushroom cultivation is a unique microbial process that can effectively bioconvert a wide range of plant polymers, including lignin, cellulose, and hemicellulose (Lakhanpal 2002). This process produces edible and nutritious mushrooms and provides an efficient and economically viable method for valorizing lignocellulosic waste materials. (Kumar et al. 2018). While there are some similarities to conventional mushroom cultivation, the cultivation of mushroom mycelium in this article is largely different. Mycelium cultivation involves growing only mycelium quickly and in large quantities while maintaining conditions that prevent the production of fruiting bodies. The waste substrate can be converted into bioenergy, biofertilizer, and biocompost, thereby reducing the environmental impact of these waste streams. (Wan Mahari et al. 2020) Furthermore, cultivation is labor-intensive and can provide employment opportunities, particularly in rural areas. (Kumar et al. 2018).

Mushroom mycelium grows radially, and becomes entangled to form a biofilm (mycelium mat). In addition, the mycelium exchanges oxygen and carbon dioxide necessary for proliferation to form aerial hyphae. The structure in which the thickness and density are increased through the growth of biofilms and aerial mycelium is called a mushroom mycelium mat. To produce a mushroom mycelium mat of high quality, optimization of the production process, including raw materials, unit operations, culture bioreactors, process controls, and economic analysis, is essential. The final mushroom mycelium products generally have lower physical strength compared to industrial counterparts. Therefore, to meet the industrial standards of each product various methods such as plasticization, cross-linking, pressing, and coating processes are employed (Chan et al. 2021; Manan et al. 2021; Yang et al. 2021; Raman et al. 2022; Elsacker et al. 2023; Teeraphantuvat et al. 2024).

Both batch and continuous processes can be explored for large-scale bioprocessing of mushroom mycelium production. In a batch process, the growth substrate is inoculated with mushroom mycelium and incubated under controlled conditions until the desired yield is achieved. In contrast, a continuous process involves the continuous feeding of growth substrate and harvesting the produced mycelium, allowing for a more efficient and scalable production system. For large-scale production of mycelium, culture substrates are sterilized and cooled in a vinyl bag, inoculated with mushroom hyphae, cultured for a certain period, and then crushed and placed in a container for mat production and secondary culturing (Gandia et al. 2022; Raman et al. 2022). In the future, automation facilities will be adopted to develop an efficient production process that shortens the time required for high-quality mushroom mycelium mat, reduces the possibility of contamination, and improves the quality to increase productivity and cost-efficiency.

There is a possibility that mycelium-based materials will fail to be commercialized. The most challenging part in the commercialization of mycelium-based materials is product homogeneity. The issue of process control, which regulates the thickness and growth direction of the mycelium, is a challenge that needs to be addressed. In addition, as the growth direction, shape, and thickness of the mycelium are varied, the mycelium content may differ depending on the sample collection location. Therefore, it is unlikely that the same amount of mycelium will be contained in each individual product, regardless of the type of composite material manufactured. This makes commercializing difficult as it is challenging to obtain the same quality amongst all products. To overcome these difficulties, the role of biochemical engineering researchers will be crucial in the future. First, it is necessary to secure culture technology that can produce mycelium in a short period, develop a device that ensures uniform material quality by controlling mycelium growth, and improve physical and biochemical properties through mixing with various materials. Additionally, cutting techniques suited to the user are required. If these technical difficulties are overcome, the transition from existing plastic-based composite materials to fungal mycelium-based composite materials is expected to be successfully achieved.

## Conclusion

Most of the materials derived from mushroom mycelium discussed in this review are replacements for existing products, meaning they must meet various performance standards in each material field. Therefore, new standards and legislation are essential for commercializing a broader range of mushroom mycelium products, and organic integration with existing production processes is necessary for developing new industrial products.

Although mushroom mycelium products have a low carbon footprint, the energy requirements for their production must be addressed. They also require significant investments in research, development, and production facilities.

Finally, we need to solve the challenges posed by fickle consumers and greenwashing in many parts of the industry to ensure that truly sustainable products can remain competitive. Academic research is essential to close the gap between research and industrialization, and we suggest that some companies make their production knowledge publicity available.

## Data Availability

Data will be made available upon request.

## Abbreviations

POD: Peroxidase
GH: Glucoside hydrolase
SEM: Scanning electron microscope
EPS: Extracellular polymeric matrix
CHS: Chitin synthase
GMO: Genetically modified organism
UV: Ultraviolet
RAPD: Random amplification of polymorphic DNA
ATMT: Agrobacterium tumefaciens-mediated transformation
NHEJ: Non-homologous end joining
DSB: DNA double-strand break
gRNA: Guide RNA
HR arm: Homologous arm sequences
HDR: Homology-direct repair
GM: Genetically modified
CWI: Cell wall integrity
GEF: Guanine nucleotide exchange factors
MPK: Mitogen-activated protein kinase
PKC: Protein kinase C
MBC: Mycelium-based composite
MBT: Mycelium-based textile
SMS: Spent mushroom substrate
PDB: Potato dextrose broth
MDD: Material-driven design
